# Extracellular Volume Fraction Derived From Dual-Layer Spectral Detector Computed Tomography for Diagnosing Cervical Lymph Nodes Metastasis in Patients With Papillary Thyroid Cancer: A Preliminary Study

**DOI:** 10.3389/fonc.2022.851244

**Published:** 2022-06-08

**Authors:** Yan Zhou, Di Geng, Guo-Yi Su, Xing-Biao Chen, Yan Si, Mei-Ping Shen, Xiao-Quan Xu, Fei-Yun Wu

**Affiliations:** ^1^ Department of Radiology, The First Affiliated Hospital of Nanjing Medical University, Nanjing, China; ^2^ Section of Clinical Research, Philips Healthcare Ltd, Shanghai, China; ^3^ Department of Thyroid Surgery, The First Affiliated Hospital of Nanjing Medical University, Nanjing, China

**Keywords:** papillary thyroid cancer, lymph node, metastasis, multidetector computed tomography, extracellular volume

## Abstract

**Objectives:**

The current study evaluates the performance of dual-energy computed tomography (DECT) derived extracellular volume (ECV) fraction based on dual-layer spectral detector CT for diagnosing cervical lymph nodes (LNs) metastasis from papillary thyroid cancer (PTC) and compares it with the value of ECV derived from conventional single-energy CT (SECT).

**Methods:**

One hundred and fifty-seven cervical LNs (81 non-metastatic and 76 metastatic) were recruited. Among them, 59 cervical LNs (27 non-metastatic and 32 metastatic) were affected by cervical root artifact on the contrast-enhanced CT images in the arterial phase. Both the SECT-derived ECV fraction (ECV_S_) and the DECT-derived ECV fraction (ECV_D_) were calculated. A Pearson correlation coefficient and a Bland–Altman analysis were performed to evaluate the correlations between ECV_D_ and ECV_S_. Receiver operator characteristic curves analysis and the Delong method were performed to assess and compare the diagnostic performance.

**Results:**

ECV_D_ correlated significantly with ECV_S_ (r = 0.925; p <0.001) with a small bias (−0.6). Metastatic LNs showed significantly higher ECV_D_ (42.41% vs 22.53%, p <0.001) and ECV_S_ (39.18% vs 25.45%, p <0.001) than non-metastatic LNs. By setting an ECV_D_ of 36.45% as the cut-off value, optimal diagnostic performance could be achieved (AUC = 0.813), which was comparable with that of ECV_S_ (cut-off value = 34.99%; AUC = 0.793) (p = 0.265). For LNs affected by cervical root artifact, ECV_D_ also showed favorable efficiency (AUC = 0.756), which was also comparable with that of ECV_S_ (AUC = 0.716) (p = 0.244).

**Conclusions:**

ECV_D_ showed a significant correlation with ECV_S_. Compared with ECV_S_, ECV_D_ showed comparable performance in diagnosing metastatic cervical LNs in PTC patients, even though the LNs were affected by cervical root artifacts on arterial phase CT.

## Introduction

Cervical lymph node (LN) metastasis plays a crucial role in the risk stratification of papillary thyroid cancer (PTC), which is associated with the determination of an individual treatment plan (1, 2). Ultrasonography (US) is the preferred imaging technique to diagnose LN metastasis in PTC patients (1–3). However, US relies on the clinical experience of the operator and is restricted to assessing the retropharyngeal and mediastinal LNs. Contrast-enhanced computed tomography (CT) can make up for this deficiency (4–6). However, based on the conventional US and CT images, we usually diagnose the LN metastasis depending on the qualitative image features, which are limited in their subjectivity (3–6). Therefore, a more accurate and quantitative method is needed.

The extracellular volume (ECV) fraction, which is usually calculated based on the conventional single-energy CT (SECT), primarily evaluates the CT value increment by subtracting unenhanced and equilibrium phase CT images (7–15). However, the requirement for unenhanced CT scans would lead to increased radiation exposure. Besides, the potential risk of misregistration between unenhanced and the equilibrium phase CT images would influence the accuracy (10, 13, 15). By contrast, dual-energy CT (DECT) could directly measure the iodine intensity based on the iodine maps in equilibrium phase (10, 15, 16). It can quantify the iodine contrast in the intravascular and extravascular–extracellular spaces without the need for co-registration. Previously, significant correlations between DECT-derived ECV fraction (ECV_D_) and SECT-derived ECV fraction (ECV_S_) have been reported in myocardial and pancreatic imaging (10, 15). As to the clinical values, ECV fraction has been proven to be useful in assessing the pathological characteristics of cardiac and hepatic fibrosis (7–13) and the clinical prognosis of some tumors (14, 15). The spread of tumor cells may destroy the inner structure, subsequently resulting in the changes in ECV fraction in the metastatic LNs from PTC (17–20). So, we hypothesized that ECV_D_ might be useful for diagnosing cervical LN metastasis from PTC.

Therefore, this study aimed to: 1) evaluate the value of ECV_D_ for diagnosing cervical LN metastasis from PTC and compare it with the value of ECV_S_; and 2) sub-group evaluate the value of ECV_D_ and ECV_S_ for diagnosing LN metastasis that is affected by cervical root artifacts on arterial phase CT.

## Materials and Methods

### Clinical Data

This prospective study was approved by the ethics committee of our institutional review board, and written informed consent was obtained from each patient. We calculated the sample size (21, 22) and prospectively recruited 110 patients with suspicious PTC for preoperative DECT evaluation from December 2020 to October 2021. The patients were routinely followed up through serology and imaging examinations (1). Inclusion criteria were as follows: 1) final diagnosis of PTC was made based on postoperative pathology; 2) cervical LN dissection was conducted and pathologically analyzed; 3) no history of other cancers; and 4) image quality was adequate for subsequent analysis. Finally, 54 patients (13 men, 41 women; mean age, 33 years; age range, 25–61 years) with PTC were enrolled in the study.

### LNs Histopathologic Labeling and Grouping

Taking the American Joint Committee on Cancer cervical regional lymph node-level system as a reference, cervical LNs were divided into 7 levels (23). According to the pathological results, cervical LNs were classified as non-metastatic or metastatic LNs. The specific process of cervical LN labeling and grouping was as follows: If the harvested LNs at one cervical level were pathologically negative, all the identified LNs on CT images at this level were grouped as non-metastatic. If the harvested LNs at one cervical level were pathologically positive, all the identified LNs on CT images at this level were grouped as metastatic. If one cervical level contained both non-metastatic and metastatic LNs, all the LNs on CT images at this level were excluded from subsequent analysis (4, 19). To reduce the risk of false “all positive” or “all negative” cervical levels, if only one LN within one specific cervical level was harvested and reported in the pathological result, this level would also be discarded. To avoid the partial volume effect, only the LNs with a maximal diameter in the short axis of more than 5 mm were enrolled in this study. Finally, 157 cervical LNs (81 non-metastatic and 76 metastatic) were analyzed.

### DECT Scanning and Post-Processing

All patients underwent neck CT examinations on a dual-layer spectral detector CT (IQon spectral CT, Philips Healthcare). Detailed acquisition parameters were as follows: tube voltage, 120 kVp; tube current, modulated by automated radiation dose control; collimation, 64 × 0.625 mm; rotation time, 0.5 s; pitch factor, 1.1. Triphasic acquisition protocol included unenhanced, early arterial phases, and equilibrium phases at 3 mm slice thickness using the spectral mode. For contrast-enhanced scanning, 75 ml of contrast agent (iopromide; Bayer HealthCare) at a flow rate of 3.5 ml/s was intravenously delivered through the right elbow vein, followed by a bolus injection of 25 ml of saline at the same flow rate. The delay times for the early arterial and equilibrium phases were fixed at 25 and 180 s after the unenhanced scan. All the original data were reconstructed into contiguous axial images with a slice thickness of 1 mm for further analyses. Post-processing workstation (IntelliSpace Protal, Version 10, Philips Healthcare) was used for image reconstruction. The 120-kVp equivalent blended images were reconstructed through an iterative algorithm (iDose^4^ [level 3], Philips Healthcare), while iodine maps were reconstructed through a spectral reconstruction algorithm (Spectral [level 3], Philips Healthcare).

To calculate radiation exposure, CT dose index (CTDIvol) and dose-length product (DLP) were recorded for each patient. The mean volume of CTDIvol and DLP using unenhanced CT images was 5.3 ± 1.1 mGy (range, 4.3–7.7 mGy) and 141.8 ± 30.9 mGy × cm (range, 117.9–199.7 mGy × cm), respectively. The mean volume CTDIvol and DLP of using dual-phase enhanced CT images was 10.4 ± 2.9 mGy (range, 8.4–14.6 mGy) and 291.5 ± 64.0 mGy × cm (range, 224.5–403.0 mGy × cm), respectively. The total mean volume of CTDIvol and DLP was 15.7 ± 3.8 mGy (range, 12.7–22.3 mGy) and 433.3 ± 94.7 mGy × cm (range, 342.4–602.7 mGy × cm), respectively.

### Qualitative Image Analysis

Qualitative image analysis was conducted by two radiologists (with 6 and 5 years of experience in head and neck radiology, respectively) who were blinded to the study design, based on the 120-kVp equivalent blended CT images in both arterial and equilibrium phase. If disagreements occurred, another radiologist (with 26 years of experience in head and neck imaging) would make the final decision. All these readers were blinded to the final clinicopathologic results. Positive findings for cervical LN metastasis were as follows: 1) a maximal diameter in short axis of ≥10 mm; 2) strong or heterogeneous enhancement was abnormal enhancement; 3) calcification; 4) cystic change; and 5) extra nodal extension, which was defined as obscure boundary or invasion into adjacent structures (4, 6). Cervical LNs affected by cervical root artifact on the contrast-enhanced CT images in the arterial phase were also recorded.

### ECV_D_ and ECV_S_ Measurement and Calculation

**Figure 1:**
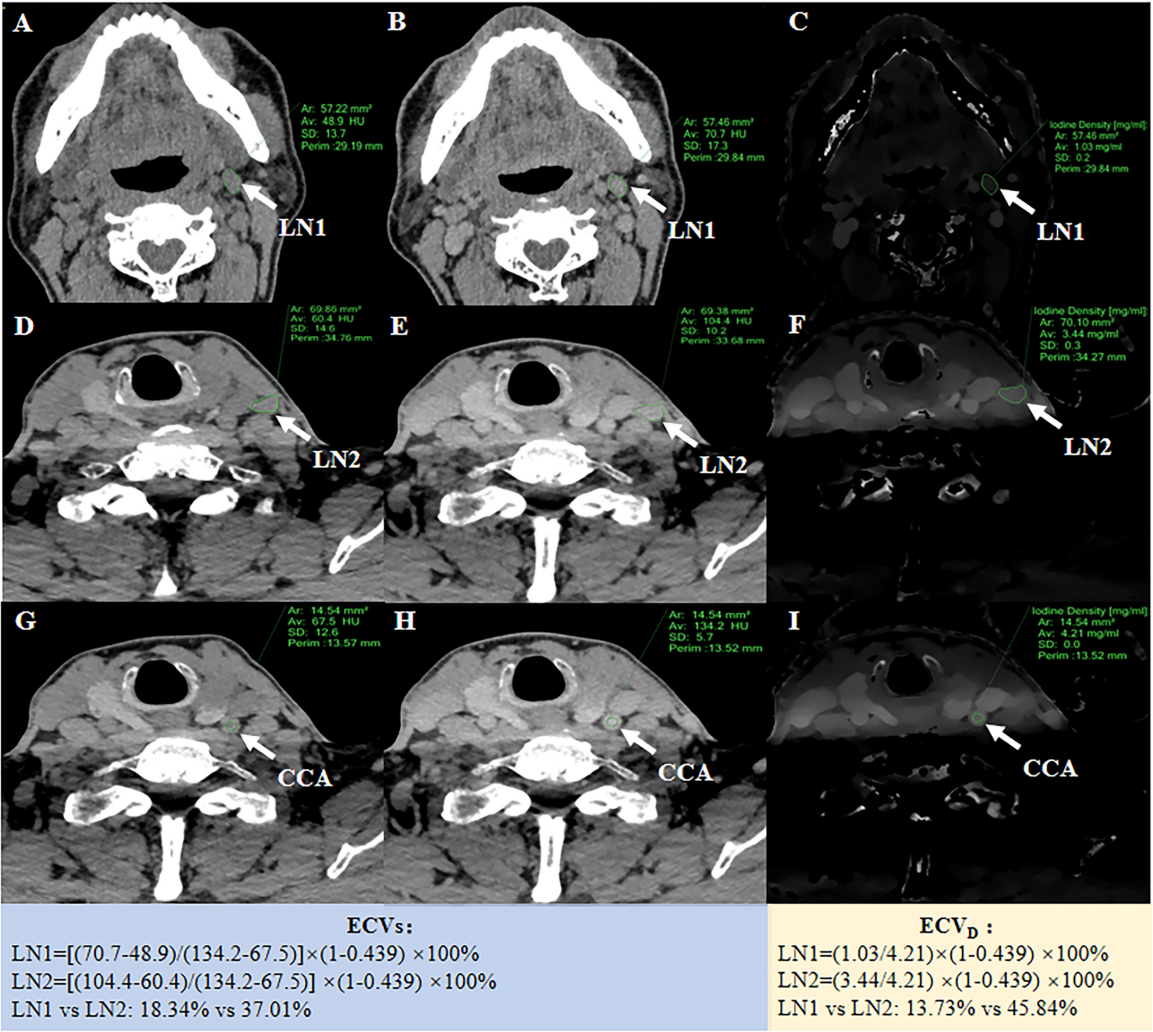
A 55-year-old PTC patient with non-metastatic LN1 in the left II level and metastatic LN2 in the left III level. The left, middle and right columns were unenhanced images **(A, D, G)**, equilibrium phase 120-kVp equivalent blended images **(B, E, H)**, and equilibrium phase iodine maps **(C, F, I)**, respectively. After ROIs were placed, quantitative parameters could be generated for LN1 and LN2, respectively. For normalization, a 15 mm^2^ circular ROI was drawn on the ipsilateral CCA. ECVs of LN1 and LN2 was 18.34% and 37.01%, respectively. ECVD of LN1 and LN2 was 13.73% and 45.84%, respectively.

Attenuation values on unenhanced and equilibrium phase 120-kVp equivalent blended images were measured for ECV_S_. Iodine density (ID) on equilibrium phase iodine maps was measured for ECV_D_. Regions of interest (ROIs) were manually drawn on the slices encompassing the whole LN. Calcification and cystic regions were excluded with reference to the unenhanced CT images. Another circular ROI (about 15 ± 5 mm^2^) was drawn on the ipsilateral common carotid artery (CCA) for normalization. ECV_S_ and ECV_D_ were calculated using the following formula: 1) ECV_S_ (%) = (1 − hematocrit) × (ΔHU_LN_/ΔHU_CCA_) ×100. ΔHU_LN_ and ΔHU_CCA_ were Hounsfield units in the equilibrium phase minus in the unenhanced phase of LN and CCA, respectively; 2) ECV_D_ (%) = (1 − hematocrit) × (ID_LN_/ID_CCA_) × 100. ID_LN_ and ID_CCA_ had iodine densities in the equilibrium phase of LN and CCA, respectively (15) (**Figure 1**). All the quantitative measurements were conducted by two radiologists (with 5 and 3 years of experience in head and neck radiology, respectively), blinded to the study design and pathological results. The average values of these two radiologists were employed for further statistical analyses.

### Statistical Analysis

Statistical analyses were performed using SPSS (version 23.0, SPSS, Chicago, IL, USA) and MedCalc (version 15.0, MedCalc, Mariakerke, Belgium) software. Sample size calculation used the method proposed by Li and Fine (21, 22). The intraclass correlation coefficient (ICC) was used to assess the inter-reader reproducibility. The agreement was interpreted as excellent (ICC >0.90), good (ICC = 0.75–0.90), moderate (ICC = 0.50–0.75), or poor (ICC <0.50) (24). Pearson correlation coefficient and Bland–Altman analysis were performed to evaluate the correlations between ECV_D_ and ECV_S_ (25). Mann–Whitney U test was conducted to compare ECV_D_ and ECV_S_ non-metastatic and metastatic cervical LNs. Receiver operating characteristic (ROC) curves analysis and the Delong method were performed to assess and compare the diagnostic efficiency of conventional qualitative LNs features and two ECV measurements (26). Optimal cutoff values were determined by maximizing the Youden index (sensitivity + specificity − 1). A two-sided *p-*value less than 0.05 was considered statistically significant.

## Results

### LN Assignment and Grouping

One hundred and fifty-seven cervical LNs (81 non-metastatic and 76 metastatic) were analyzed. The distribution of all 81 non-metastatic LNs was as follows: 1) II level with 30 LNs; 2) III level with 17 LNs; 3) IV level with 9 LNs; and 4) VI level with 25 LNs. The distribution of all 76 metastatic LNs was as follows: 1) II level with 5 LNs; 2) III level with 7 LNs; 3) IV level with 20 LNs; and 4) V level with 1 LN; 5) VI level with 43 LNs. The mean size of all 81 non-metastatic and 76 metastatic LNs was 8.4 ± 1.3 mm and 14.2 ± 2.7 mm, respectively (**Table 1**).

**Table 1:** Conventional qualitative features for diagnosing metastatic lymph nodes.

Features	Total LNs(157)		Cervical root artifact affected LNs (59)	
	Non-metastatic LNs (81)	Metastatic LNs (76)	Non-metastatic LNs (27)	Metastatic LNs (32)
Size (mm)	8.4 ± 1.3	14.2 ± 2.7	7.1 ± 0.8	11.3 ± 1.2
Location				
II	30 (37.0%)	5 (6.6%)	0 (0%)	0 (0%)
III	17 (21.0%)	7 (9.2%)	0 (0%)	0 (0%)
IV	9 (11.1%)	20 (26.3%)	2 (7.4%)	4 (12.5%)
V	0 (0.0%)	1 (1.3%)	0 (0%)	0 (0%)
VI	25 (30.9%)	43 (56.6%)	25 (92.6%)	28 (87.5%)
Negative findings	74 (91.4%)	23 (30.3%)	22 (81.5%)	17 (53.1%)
Positive findings	7 (8.6%)	53 (69.7%)	5 (18.5%)	15 (46.9%)
Size **>**10 mm	1 (1.2%)	22 (28.9%)	0 (0.0%)	3 (9.4%)
Abnormal enhancement	6 (7.4%)	51 (67.1%)	5 (18.5%)	15 (46.9%)
Calcification	0 (0.0%)	7 (9.2%)	0 (0.0%)	1 (3.1%)
Cystic change	0 (0.0%)	8 (10.5%)	0 (0.0%)	0 (0.0%)
Extra nodal extension	0 (0.0%)	1 (1.3%)	0 (0.0%)	0 (0.0%)

Size is expressed as mean ± standard deviation. Other data are numbers of lymph nodes and parentheses indicate the proportion.

LNs, lymph nodes.

Fifty-nine cervical LNs (27 non-metastatic and 32 metastatic) were affected by cervical root artifact on arterial phase CT. The distribution of all 27 non-metastatic LNs was as follows: 1) IV level with 2 LNs; and 2) VI level with 25 LNs. The distribution of all 32 metastatic LNs was as follows: 1) IV level with 4 LNs; and 2) VI level with 28 LNs. The mean size of all 27 non-metastatic and 32 metastatic LNs was 7.1 ± 0.8 mm and 11.3 ± 1.2 mm, respectively (**Table 1**).

### Conventional Qualitative LN Features

Positive findings of size >10 mm, abnormal enhancement, calcification, cystic change, or extra nodal extension were 69.7% (53 of 76) in all metastatic cervical LNs, while 46.9% (15 of 32) were affected by cervical root artifacts on arterial phase CT. Detailed conventional qualitative features for diagnosing metastatic cervical LNs are summarized in **Table 1**. The diagnostic performance of these conventional qualitative LN features is summarized in **Table 2**.

**Table 2:** Diagnostic performance of conventional CT image features for diagnosing metastatic lymph nodes.

	AUC	Sensitivity	Specificity	PPV	NPV
Total LNs					
Size >10 mm	0.638 (0.556, 0.713)	0.588 (0.488, 0.706)	0.687 (0.633, 0.700)	0.755 (0.572, 0.799)	0.606 (0.517, 0.690)
Abnormal enhancement	0.692 (0.619, 0.753)	0.658 (0.537, 0.765)	0.726 (0.646, 0.772)	0.750 (0.653, 0.831)	0.689 (0.574, 0.758)
Calcification	0.548 (0.466, 0.628)	0.096 (0.039, 0.190)	1.000 (0.955, 1.000)	1.000 (0.590, 1.000)	0.551 (0.467, 0.633)
Cystic change	0.555 (0.473, 0.635)	0.110 (0.049, 0.205)	1.000 (0.955, 1.000)	1.000 (0.631, 1.000)	0.555 (0.470, 0.637)
Extra nodal extension	0.507 (0.425, 0.588)	0.014 (0.001, 0.074)	1.000 (0.955, 1.000)	1.000 (0.025, 1.000)	0.529 (0.447, 0.611)
Cervical root artifact affected LNs					
Size >10 mm	0.560 (0.416, 0.697)	0.436 (0.390, 0.599)	0.750 (0.703, 0.791)	0.793 (0.613, 0.839)	0.551 (0.402, 0.693)
Abnormal enhancement	0.647 (0.513, 0.772)	0.613 (0.403, 0.802)	0.701 (0.509, 0.811)	0.712 (0.445, 0.883)	0.637 (0.455, 0.799)
Calcification	0.520 (0.377, 0.661)	0.040 (0.001, 0.204)	1.000 (0.872, 1.000)	1.000 (0.025, 1.000)	0.529 (0.385, 0.671)
Cystic change	NA	NA	NA	NA	NA
Extra nodal extension	NA	NA	NA	NA	NA

Data in parentheses indicate 95% confidence interval.

LNs, lymph nodes; PPV, positive predictive value; NPV, negative predictive value; NA, not applicable.

### ECV_D_ and ECV_S_ Correlation

**Figure 2:**
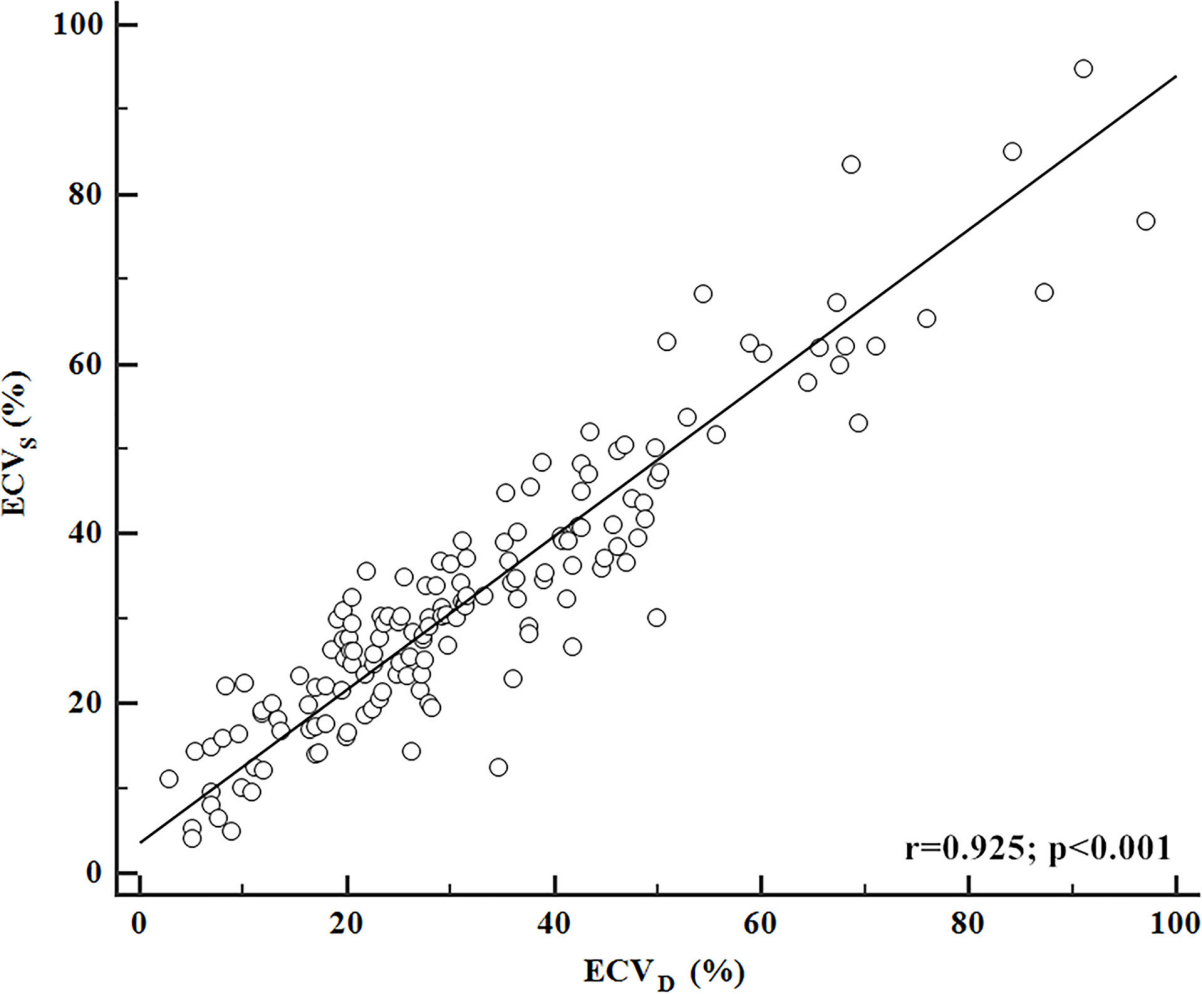
Correlation between ECV_D_ and ECV_S_. ECV_D_ showed a strong positive correlation with ECV_S_ (r = 0.925; p <0.001).

**Figure 3:**
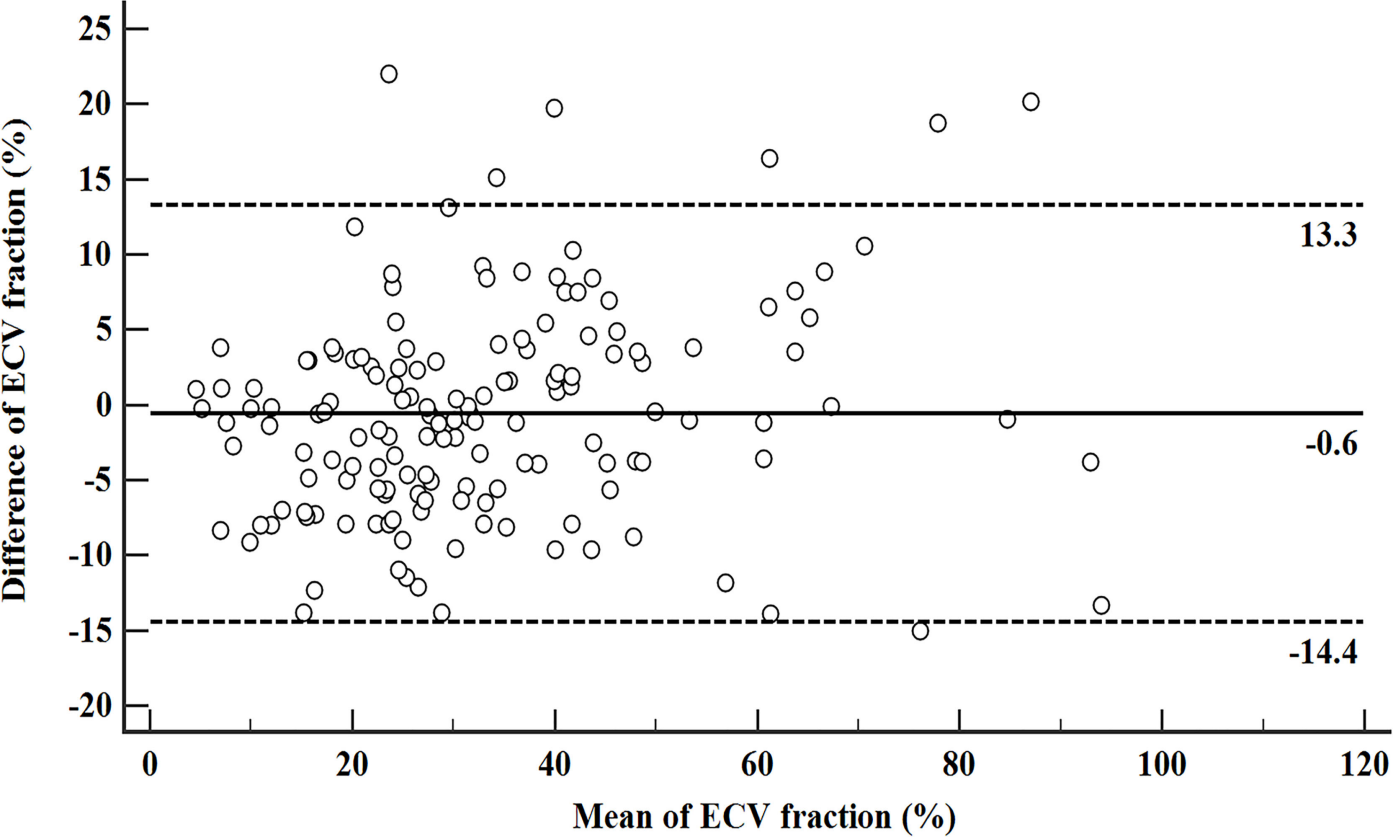
Bland–Altman plot for ECV_D_ and ECV_S_. The solid line stands for mean bias (−0.6), and the lower and upper dotted lines stand for 95% limits of agreement (−14.4 and 13.3).

The inter-reader agreement in the measurement of ECV_S_ and ECV_D_ was excellent with an ICC of 0.929 [95% confidence interval (CI): 0.893, 0.978] and 0.902 (95% CI: 0.878, 0.946), respectively. A strong positive correlation was found between ECV_D_ and ECV_S_ [r = 0.925 (95% CI: 0.899, 0.945); p <0.001). A scatter plot between ECV_D_ and ECV_S_ is shown in **Figure 2**. The Bland–Altman plot for ECV_D_ and ECV_S_ showed a small bias (−0.6) with the lower and upper limits of agreement of −14.4 and 13.3 (**Figure 3**).

### Diagnostic Performance and Comparison of ECV_D_ and ECV_S_

**Figure 4:**
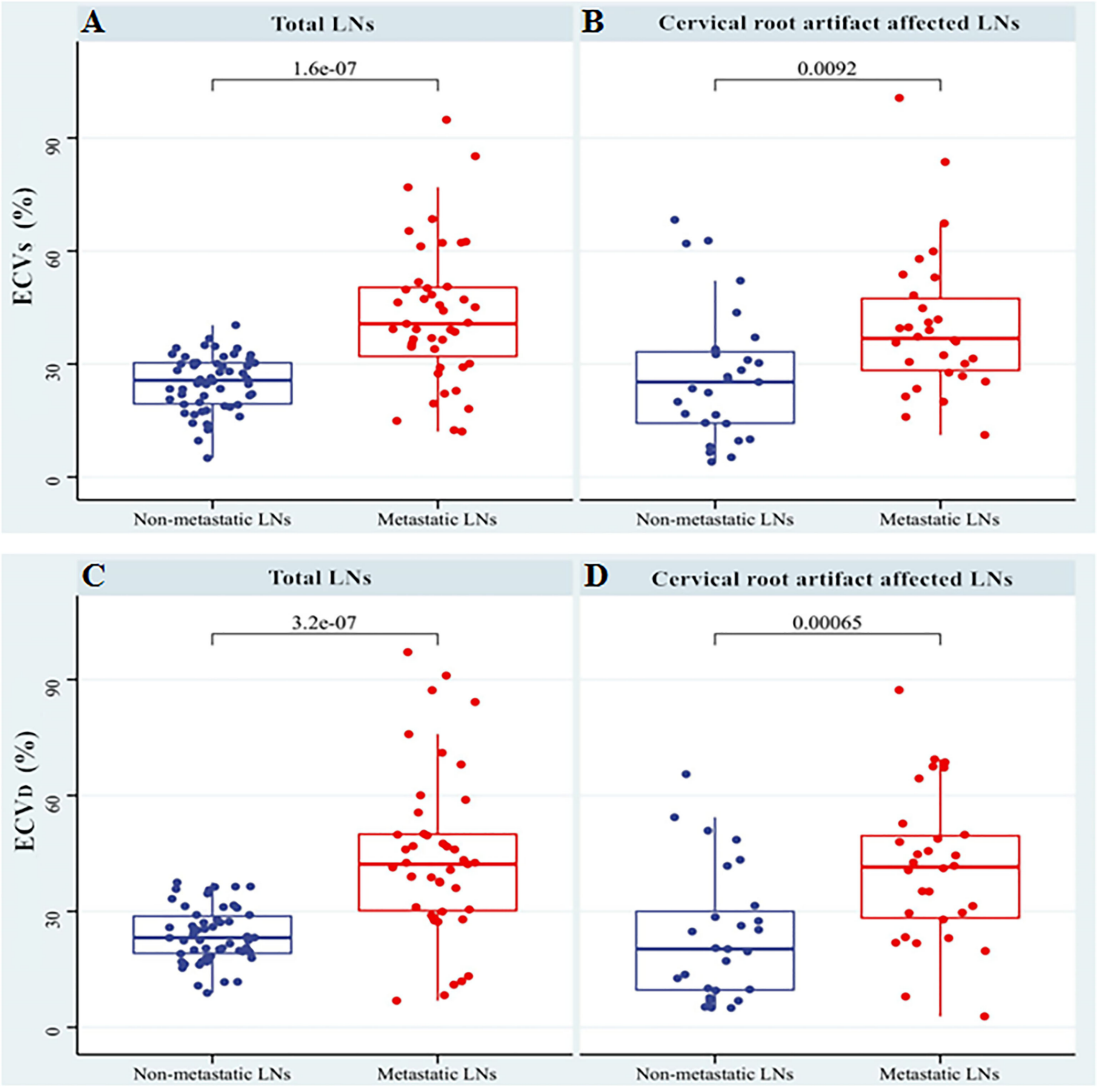
Box plots of ECV_S_ and ECV_D_ between non-metastatic and metastatic LNs groups in total LNs **(A, C)** and the LNs affected by cervical root artifact on arterial phase CT **(B, D)**.

**Figure 5:**
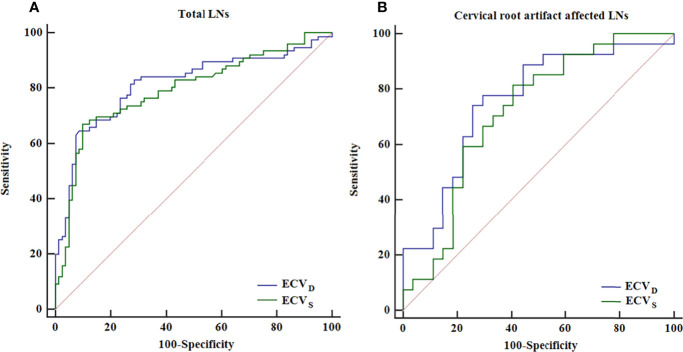
ROC curve analysis of ECV_S_ and ECV_D_ for diagnosing total LNs **(A)** and the LNs affected by cervical root artifact on arterial phase CT **(B)**.

In the non-metastatic LN group, ECV_S_ and ECV_D_ were 25.45% [interquartile range (IQR): 18.15%, 31.17%] and 22.53% (IQR: 16.97%, 29.09%), respectively. In the metastatic LN group, ECV_S_ and ECV_D_ were 39.18% (IQR: 30.09%, 50.44%) and 42.41% (IQR: 29.58%, 54.90%), respectively. Metastatic LNs showed significantly higher ECV_D_ (42.41% vs 22.53%, p <0.001) and ECV_S_ (39.18% vs 25.45%, p <0.001) than non-metastatic LNs. Box plots of two ECV fractions between the non-metastatic and metastatic LNs groups are shown in **Figures 4A, C**. Setting an ECV_D_ of 36.45% as the cut-off value, optimal diagnostic performance could be achieved with an AUC of 0.813 (95% CI: 0.743, 0.871), which was comparable with that of ECV_S_ [cut-off value = 34.99%; AUC = 0.793 (95% CI: 0.721, 0.853); p = 0.265]. The two ECV measurements both performed significantly better than conventional qualitative LN features (p all >0.05). The detailed diagnostic performance of ECV_S_ and ECV_D_ is summarized in **Table 3** and shown in **Figure 5A**.

**Table 3:** ECV_S_ and ECV_D_ for diagnosing metastatic lymph nodes.

	ECV_S_ (%)				ECV_D_ (%)			
	Total LNs(157)		Cervical root artifact affected LNs (59)		Total LNs (157)		Cervical root artifact affected LNs (59)	
	Non-metastatic LNs (81)	Metastatic LNs (76)	Non-metastatic LNs (27)	Metastatic LNs (32)	Non-metastatic LNs (81)	Metastatic LNs (76)	Non-metastatic LNs (27)	Metastatic LNs (32)
ECV (%)	25.45 (18.15–31.17)	39.18 (30.09–50.44)	25.23 (14.20–33.93)	35.96 (27.74–41.83)	22.53 (16.97–29.09)	42.41 (29.58–54.90)	20.27 (9.53–31.48)	41.19 (27.90–49.85)
*p*-value	<0.001		<0.001		<0.001		<0.001	
Cut-off Value (%)	34.99		26.63		36.45		27.52	
AUC	0.793 (0.721, 0.853)		0.716 (0.577, 0.830)		0.813 (0.743, 0.871)		0.756 (0.620, 0.862)	
Sensitivity	0.671 (0.554, 0.775)		0.815 (0.619, 0.937)		0.645 (0.527, 0.751)		0.778 (0.577, 0.914)	
Specificity	0.901 (0.815, 0.956)		0.593 (0.388, 0.776)		0.914 (0.830, 0.965)		0.704 (0.498, 0.862)	
PPV	0.864 (0.750, 0.940)		0.667 (0.482, 0.820)		0.875 (0.759, 0.948)		0.724 (0.528, 0.873)	
NPV	0.745 (0.647, 0.828)		0.762 (0.528, 0.918)		0.733 (0.635, 0.816)		0.760 (0.549, 0.906)	

ECV is expressed as median with interquartile range in parentheses. Other data in parentheses indicate 95% confidence interval.

LNs, lymph nodes; ECV, extracellular volume; ECV_S_, SECT-derived ECV fraction; ECV_D_, DECT-derived ECV fraction; PPV, positive predictive value; NPV, negative predictive value.

### LNs Affected by Cervical Root Artifact

**Figure 6:**
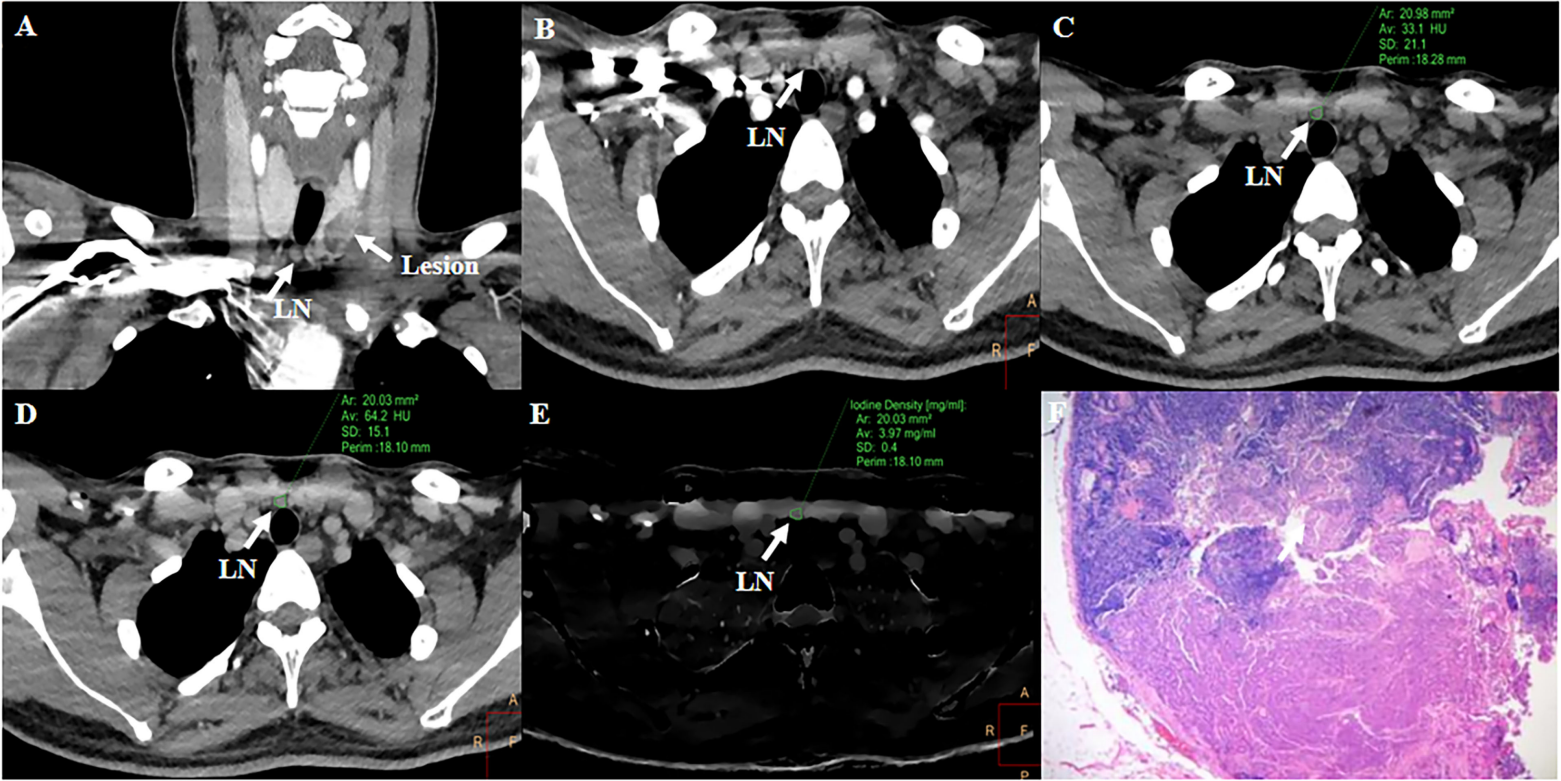
Representative case of using ECV_S_ and ECV_D_ to diagnose LN metastasis affected by cervical root artifact on arterial phase CT. A 36-year-old PTC patient with a LN in the right VI level. Right cervical root artifact existed coronal **(A)** and axial **(B)** early arterial phase CT images. Right cervical root artifact was reduced in equilibrium phase images. Attenuation values and iodine density were measured in the unenhanced **(C)**, equilibrium phase 120-kVp equivalent blended image **(D)**, and iodine map **(E)**. The ECV_S_ and ECV_D_ of the LN were 50.32 and 53.15%, respectively. Subsequent pathological examination confirmed this LN as metastatic **(F)**.

For cervical LNs affected by cervical root artifact on the arterial phase contrast-enhanced CT images, a significantly higher ECV_D_ (41.19% vs 20.27%, p <0.001) and ECV_S_ (35.96% vs 25.23%, p <0.001) of metastatic LNs than non-metastatic were also found (**Figures 4B, D**). ECV_D_ also showed good efficiency with an AUC of 0.756 (95% CI: 0.620, 0.862), which was also comparable with that of ECV_S_ [AUC = 0.716 (95% CI: 0.577, 0.830); p = 0.244]. Detailed diagnostic efficiency of ECV_S_ and ECV_D_ is summarized in **Table 3** and shown in **Figure 5B**. A representative case of using ECV_S_ and ECV_D_ for diagnosing cervical LNs affected by cervical root artifact is shown in **Figure 6**.

## Discussion

In our study, we found that both the ECV_D_ and ECV_S_ of metastatic cervical LNs were significantly higher than those of non-metastatic cervical LNs in PTC patients. A significant positive correlation was found between the two ECV fractions. Besides, compared with ECV_S_, ECV_D_ showed comparable performance in diagnosing metastatic cervical LNs, even though the LNs were affected by cervical root artifact on arterial phase CT images.

Morphological features of LNs, namely, enlarged size, abnormal enhancement, calcification, cystic change, and extra nodal extension, were reported to be useful in diagnosing metastatic cervical LNs in PTC patients (4, 6, 19). However, one major disadvantage of the morphological features was their subjectivity. Besides, the proportion of the metastatic cervical LNs showing abnormal morphological CT features was not high. In this study, only 69.7% (53/76) of metastatic cervical LNs exhibited positive findings for morphological CT features. In our opinion, the relatively low rate of abnormal morphological CT features might be due to the high proportion of small cervical LN metastasis in PTC patients (1, 2). Among these features, strong or heterogeneous enhancement on early arterial phase CT was reported to be associated with metastatic LNs (4, 19, 27). However, contrast-enhanced CT images in the arterial phase were prone to being influenced by the unavoidable cervical root artifact, which might negatively affect the evaluation of enhancement characteristics (28, 29). In the subgroup analysis of the LNs affected by cervical root artifact in our study, positive morphological CT features were found only in 46.9% (15/32) of PTC patients. Therefore, one more sensitive and reliable method for diagnosing metastatic LNs from PTC is needed.

ECV fraction was reported to be a promising quantitative metric in evaluating cardiac and hepatic fibrosis, and the prognosis of some tumors (7–15). In our study, metastatic cervical LNs demonstrated significantly higher ECV_D_ and ECV_S_ than non-metastatic LNs in PTC patients. The normal structure of LNs contains numerous lymphocytes in the cortex. The intensive distribution of lymphocytes in normal LN results in relatively smaller intravascular and extravascular–extracellular spaces (30). In contrast, the disseminated tumor cells might destruct the normal structure of LNs, subsequently resulting in enlarged intravascular and extravascular–extracellular spaces (17–20). Enlarged intravascular and extravascular–extracellular spaces could store more contrast agents in the equilibrium phase, which might lead to the increased ECV fractions of metastatic cervical LNs in PTC patients.

Similar to previous studies (10, 15), ECV_D_ was positively correlated with ECV_S_ in our study. Both these two ECV fractions had good performance in diagnosing metastatic LNs. Compared with ECV_S_, ECV_D_ showed comparable or even better diagnostic performance, although the difference did not reach statistical significance. As previous studies indicated (31–33), compared with SECT-derived CT values, the DECT-derived quantitative iodine contrast concentration could more effectively and reliably detect the subtle enhancement within tissues. Besides, when ECV_D_ was applied, the radiation exposure could be reduced by omitting the unenhanced CT scan (8, 13). In our study, CTDIvol was decreased from 15.7 to 10.4 mGy (66.2%), and DLP was decreased from 433.3 to 291.5 mGy × cm (67.3%), using ECV_D_ instead of ECV_S_. In our opinion, these were two advantages when ECV_D_ was used.

Previous studies have recommended an early arterial phase (25-second delay) CT scan for detecting metastatic LNs from PTC (4, 27). They found maximum differences in CT values between non-metastatic and metastatic LNs in the early arterial phase (4). However, the high concentration contrast medium within the subclavian vein at early arterial phase CT images might cause unavoidable artifacts of hardening wiring harness in the cervical root (28, 29). As we know, PTC is prone to the occurrence of LN metastasis at level IV and VI regions (1). In this situation, diagnoses of LN metastasis located in these regions might be affected by cervical root artifacts on arterial phase CT images. With the prolongation of scanning delay time, intravenous contrast medium dramatically decreased, and cervical root artifacts were also alleviated. For this reason, ECV fractions based on the equilibrium phase scan still possessed good diagnostic efficiency in the sub-group analysis of our study.

This study had several limitations. Firstly, during the calculation of sample size (setting test level as 0.05 and test power as 0.8) according to a previous method (21, 22), a sample size of 67 patients (176 LNs) was needed. However, the sample size of this preliminary study (54 patients with 157 LNs) was smaller than that calculated. This may decrease the statistical power and affect the ability of our results to reaching a sound conclusion. A larger sample size is needed to validate these results. Secondly, this study recruited PTC patients from December 2020 to October 2021. The follow-up was not long. Third, the retropharyngeal LNs, mediastinal LNs, and posterior sternal LNs were not enrolled in and assessed in our study. Fourth, 120-kVp equivalent blended images were not the same as the conventional 120-kVp CT images. This situation might have a minimal influence on the attenuation measurement. Fifth, the diagnostic performance between US and CT was not compared in our study. A comparative study on diagnostic efficacy between different imaging modalities is needed. Sixth, the application of an equilibrium phase scan might increase the radiation dose to a certain extent. In the future, an effective method for reducing the radiation dose is needed (34, 35).

In conclusion, a significant positive correlation was found between ECV_D_ and ECVs in our study. Compared with ECV_S_, ECV_D_ could provide comparable efficiency in diagnosing cervical LN metastasis in PTC patients, even though the LNs are affected by cervical root artifacts on arterial phase CT.

## Data Availability Statement

The original contributions presented in the study are included in the article/supplementary material. Further inquiries can be directed to the corresponding authors.

## Ethics Statement

The First Affiliated Hospital of Nanjing Medical University. The patients/participants provided their written informed consent to participate in this study. Written informed consent was obtained from the individual(s) for the publication of any potentially identifiable images or data included in this article.

## Author Contributions

YZ, DG, GYS, X-QX, and F-YW contributed to the conceptualization of the study. YZ, DG, G-YS, and X-QX interpreted and analyzed the data. YZ and DG performed the statistical analysis. XBC provided software and methodology support. YS and MPS conducted clinical supervision. YZ wrote original draft. X-QX and F-YW reviewed and edited the draft. F-YW provided the funding support and projected administration. All authors listed have made a substantial, direct, and intellectual contribution to the work and approved it for publication.

## Funding

This study received funding by the Natural Science Foundation of China (82171928) and the Natural Science Foundation of Jiangsu Province (BK20201494).

## Conflict of Interest

Author X-BC was employed by Philips Healthcare.

The remaining authors declare that the research was conducted in the absence of any commercial or financial relationships that could be construed as a potential conflict of interest.

## Publisher’s Note

All claims expressed in this article are solely those of the authors and do not necessarily represent those of their affiliated organizations, or those of the publisher, the editors and the reviewers. Any product that may be evaluated in this article, or claim that may be made by its manufacturer, is not guaranteed or endorsed by the publisher.

## Abbreviations

LN, Lymph node; PTC, Papillary thyroid cancer; US, Ultrasonography; ECV, Extracellular volume; CT, Computed Tomography; DECT, Dual-energy CT; SECT, Single-energy CT; ECV_S_, SECT-derived ECV fraction; ECV_D_, DECT-derived ECV fraction; CTDIvol, CT dose index; DLP, Dose-length product; ROC, Receiver operating characteristic; ID, Iodine density; ROI, Region of interest; CCA, Common carotid artery; ICC, Intraclass correlation coefficient; CI, Confidence interval; IQR, Interquartile range.
